# CYNTENATOR: Progressive Gene Order Alignment of 17 Vertebrate Genomes

**DOI:** 10.1371/journal.pone.0008861

**Published:** 2010-01-28

**Authors:** Christian Rödelsperger, Christoph Dieterich

**Affiliations:** 1 Institute for Medical Genetics, Charité-Universitätsmedizin, Berlin, Germany; 2 Max Planck Institute for Molecular Genetics, Berlin, Germany; 3 Bioinformatics in Quantitative Biology, Berlin Institute for Medical Systems Biology, Berlin, Germany; University of Pennsylvania School of Medicine, United States of America

## Abstract

Whole genome gene order evolution in higher eukaryotes was initially considered as a random process. Gene order conservation or conserved synteny was seen as a feature of common descent and did not imply the existence of functional constraints. This view had to be revised in the light of results from sequencing dozens of vertebrate genomes.

It became apparent that other factors exist that constrain gene order in some genomic regions over long evolutionary time periods. Outside of these regions, genomes diverge more rapidly in terms of gene content and order.

We have developed CYNTENATOR, a progressive gene order alignment software, to identify genomic regions of conserved synteny over a large set of diverging species. CYNTENATOR does not depend on nucleotide-level alignments and a priori homology assignment. Our software implements an improved scoring function that utilizes the underlying phylogeny.

In this manuscript, we report on our progressive gene order alignment approach, a and give a comparison to previous software and an analysis of 17 vertebrate genomes for conservation in gene order.

CYNTENATOR has a runtime complexity of 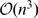 and a space complexity of 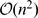 with 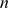 being the gene number in a genome. CYNTENATOR performs as good as state-of-the-art software on simulated pairwise gene order comparisons, but is the only algorithm that works in practice for aligning dozens of vertebrate-sized gene orders.

Lineage-specific characterization of gene order across 17 vertebrate genomes revealed mechanisms for maintaining conserved synteny such as enhancers and coregulation by bidirectional promoters. Genes outside conserved synteny blocks show enrichments for genes involved in responses to external stimuli, stimuli such as immunity and olfactory response in primate genome comparisons. We even see significant gene ontology term enrichments for breakpoint regions of ancestral nodes close to the root of the phylogeny. Additionally, our analysis of transposable elements has revealed a significant accumulation of LINE-1 elements in mammalian breakpoint regions. In summary, CYNTENATOR is a flexible and scalable tool for the identification of conserved gene orders across multiple species over long evolutionary distances.

## Introduction

Whole genome evolution operates on different levels of detail: from single nucleotides to functional elements (e.g. genes) to whole chromosomes [1]. An interesting phenomenon in the evolution of whole genomes is the existence of conserved synteny, which is the maintenance of gene content and order in certain chromosomal regions of two or more related species. Ever since Nadeau and Taylor [2] published their groundbreaking paper on the distribution of synteny breakpoints in the human and mouse genome, it was commonly believed that breakpoints are essentially distributed at random. In other words, gene order conservation is a feature of common descent and does not imply the existence of functional constraints, which would preserve gene orders. With the advent of whole genome sequencing, this view is increasingly challenged by hard data. For example, several invertebrate genomes contain operons (e.g. nematodes [3] and ascidians [4]), where gene order is functionally constrained by the necessity to generate a poly-cistronic messenger RNA. Pevzner and Tesler [5] were the first to report a deviation from the “random” breakpoint model for vertebrates. They distinguish “fragile” from “solid” regions. Fragile regions accumulate breakpoints whereas solid regions remain intact over long evolutionary periods. Several genome-wide studies highlighted potential explanations for the existence of regions of conserved synteny in distantly related genomes (e.g. [6]). Long-ranging mechanisms of gene regulation are a recurring theme in this context. Especially single developmental genes are often found in regions of conserved synteny [7]. Kikuta et al. [8] demonstrated that interspersed regulatory elements, which control the expression of such genes, are often located in introns of surrounding genes (bystander genes). This configuration cannot be broken up without a loss of regulatory inputs and constitutes a functional constraint on genome rearrangement. Another simple constrained scenario arises from bidirectional gene pairs, which share a common promoter [9].

These two examples illustrate how analysis of conserved synteny might provide insights into the evolution of regulatory mechanisms and biological functions.

### Previous Work

We and others have presented several approaches for the identification of conserved syntenic regions, which can be grouped into two classes: The first class uses ideas from set theory to identify maximal gene clusters, which fulfill certain criteria in terms of gene-gene distance, orientation and orthology relations. Such approaches have been implemented in the TEAM software [10], ADHoRe [11], LineUp [12], the Max-gap Clusters by Multiple Sequence Comparison (MCMuSeC) [13] and more generically in a correspondance multigraph approach termed cccpart [14]. The program OrthoCluster [15] is another development in this domain. OrthoCluster implements several combinations of side constraints for the identification of conserved gene clusters. It combines a set enumeration tree strategy with an efficient search on this tree to detect orthologous gene clusters in multiple genomes for a predefined seed window size. It has to be noted that these approaches identify cooccurring gene clusters that are not restricted on colinearity which is the case in our definition of conserved synteny.

A second class consists of programs like ColinearScan [16], DAGchainer [17], FISH [18], and SyMAP [19], which employ dynamic programming to detect pairwise conserved gene orders. Recently, we developed the SYNTENATOR software [20], which uses dynamic programming in combination with a partial order graph representation to detect conserved gene orders in multiple genomes. Table 1 gives an overview of the described approaches. Some of the mentioned programs are theoretically capable to perform multiple genome comparisons [13], [15], [20], but in practice they exceed acceptable costs in terms of memory and computation time as soon as they are confronted with a large number of vertebrate genomes.

**Table 1:** Overview of synteny prediction methods.

Software	Reference	Homology type	Strandedness	Colinearity	Clustering	Genomes
MCMuSeC	[13]	binary	−	+	+	*
OrthoCluster	[15]	binary	+/−	+	+	*
Cynteny	[40]	binary	+	+	−	N
cccpart	[14]	binary	−	+	+	N
LineUp	[12]	binary	−	+	+	2
TEAM	[10]	binary 1∶1	−	+	+	N
ADHoRe	[11]	binary	+	+	−	2
FISH	[18]	binary	−	+	−	2
DAGchainer	[17]	gene-specific	−	+	−	2
SyMAP	[19]	gene-specific	−	+	−	2
ColinearScan	[16]	binary	−	+	−	2
Syntenator	[20]	gene-specific	+	+	−	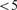
CYNTENATOR		gene-specific	+	+	−	N

Existing methods for identification of conserved syntenic regions differ in many criteria like the type of the homology data used, strand awareness and gene order conservation (colinearity/clustering, whereby colinearity implies clustering). The ‘Homology type’ column indicates how matches between genes are scored and what kind of homology data is used, ‘binary 1∶1’ denotes for example best-reciprocal hits and ‘binary’ indicates that some kind of binary gene family concept like COGs, Inparanoid or EnsEMBL can be used. ‘gene-specific’ means that BLASTP similarities or conserved distances are used in the scoring function. Of all the listed approaches, OrthoCluster is the most flexible. ‘*’ For the two most recent approaches (OrthoCluster and MCMuSeC), computation of 17 vertebrate genome comparisons proved to be not feasable.

We propose a method, called CYNTENATOR, to discover conserved syntenic regions over large evolutionary distances by progressive multiple gene order alignment. A key feature of our approach is its dynamic integration of protein-level similarities and gene context. Consequently, we do not need to assign homology relations to genes in the first place. This method is rooted in our SYNTENATOR approach for detecting conserved gene orders [20] and scales, unlike SYNTENATOR, to dozens of vertebrate genomes (17 in this study). We improved on the efficiency of our approach by recasting it into a profile-profile alignment setting, which is an extension of the Waterman-Eggert algorithm [21] to the comparison of multiple gene orders. We enhanced our scoring function to explicitly consider the phylogenetic distance of each gene pair in the sum-of-pair scoring scheme.

## Methods

### Pairwise Gene Order Alignments

We employ a similar approach as our previous software SYNTENATOR [20]. The basic concept is to compute alignments between sequences where the alphabet consists of genes rather than nucleotides or amino acids. Chromosomes are represented as linear sequences of genes and homologies between genes are defined by the bitscores from all vs. all BLASTP searches [22] among all species of interest. In a pairwise comparison Smith-Waterman local alignments [23] are computed between all chromosomes or contigs and a modified backtracking strategy is employed to extracted all non-intersecting local alignments with a score higher than a predefined threshold. This is identical to our previously published work [20].

**Figure 1:**
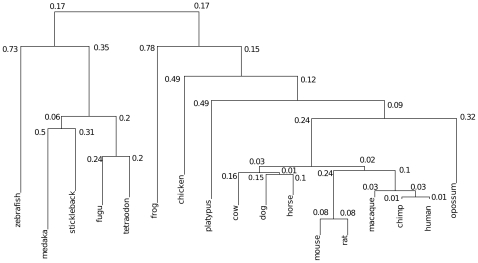
Phlyogenetic tree of 17 vertebrates. Dendroscope view on a subtree of the 28-way MULTIZ alignment tree [24], [38] which we used as a guide tree for the progressive alignment, carried out by CYNTENATOR. Distances at branches indicate the average number of substitutions per site in blastz alignments [39]. These distances were used to weight the scores between gene matches in the alignment.

A match between two genes 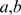 is computed from the pairwise bitscores of BLAST similarities [22] and the distances in a species tree for 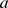 and 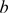 (Figure 1).
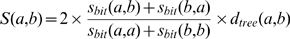
(1)Mismatch, linear gap, and minimal alignment score threshold are adjusted at each step by multiplying with 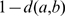 whereby 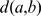 denotes the phylogenetic distance between both species. The factor of 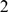 is a reminiscent of SYNTENATOR and is meant to adapt the matchscores of both programs to a comparable level.

### Progressive Alignment Procedure

For multiple genome comparisons, a guide tree is used to determine the alignment order. Single genomes correspond to leaf nodes and pairwise alignments to their parent nodes. Inner nodes can either be aligned to a leaf node or to another inner node (profile-profile alignment). Matches between two positions of multiple alignments 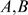 are scored using a sum of pairs score.
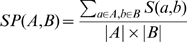
(2)


For the sum of pair scoring we multiplied mismatch, linear gap, and minimal alignment score thresholds with 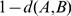 (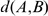 denotes the phylogenetic distance between the species in A and B). In short, missing homologous gene pairs of two closely related species are penalized more severly than missing gene pairs of two remotely related species.

The progressive alignment methodology translates to a runtime complexity of 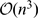 and a space complexity of 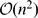 with 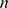 being the gene number in a given genome.

### Alignment Filters

Pairwise and multiple alignments of vertebrate genomes may result in hundreds to thousands of local alignments. We implemented several filters to lower the computational costs and the degree of redundancy among the alignments. First, we discard all alignments or single genome regions that were used to compute the alignments for the current guide tree node. For example, for a comparison of the human, mouse, and rat genomes, first mouse and rat are aligned. Only the mouse-rat pairwise alignments are used for comparisons with the human genome and all other sequence regions from mouse and rat are discarded. Second, all alignments are ordered and processed by decreasing score. We start with the highest scoring alignment and retain all alignments that do not overlap with gene sets from higher scoring alignments. Optionally, more alignments could be retained from the original ordered list, if the total number of alignments does not exceed a user defined threshold (default is 1000) and if any gene in the given alignment occurred less then 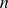 times in higher scoring alignments (gene coverage; default is 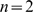). These additional alignments would contain information about paralogous conserved syntenic regions.

Within the filtering procedure, the gene-specific scoring plays a crucial role in distinguishing paralagous gene clusters of equal length. Since alignments can be ranked, correct assignments will be saved as unique alignments in the first filtering step (Figure **S1**). Another available filter singles out alignments under a minimal length.

Based on comparisons between human and mouse, we examined the effect of the gene coverage parameter on the number of aligned gene pairs. With the default value of 2 we already detect 90% of the gene pairs that may be obtained when increasing this filter parameter to 7 (Figure **S2**).

In summary, the progressive alignment procedure for multiple genome comparison, the phylogenetic adjustment of the scoring between genes, and the possibility of retaining alignments of paralogous loci at each ancestral node in the guide tree are the three major improvements over our previous software SYNTENATOR [20].

### Simulation

**Figure 2:**
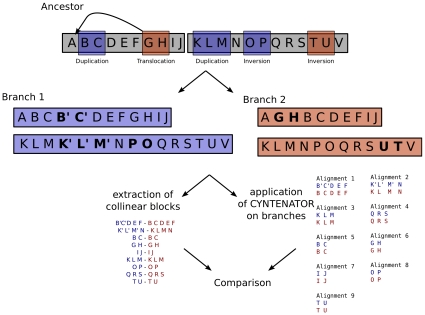
Simulation model of speciation events. We used a naive model for speciation events to create some test sets. In this example, the ancestor genome consists of two chromosomes with genes 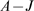 and 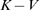. We copy this genome and apply to each branch a number of independent rearrangements. Knowing the evolutionary history of the two branches we can extract all perfect colinear blocks as regions between breakpoints. According to the mapping of genes, homology data is created and passed to CYNTENATOR together with the gene annotations of the branches (see Methods). The CYNTENATOR alignments can then be compared to the simulated blocks.

To evaluate the performance of different software and strategies on detecting conserved syntenic regions, we created a simple synthetic scenario of genome evolution (see Figure 2): 1) We generated a small genome with 1040 genes, which are distributed over 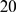 chromosomes 2) We evolved this genome twenty times independently by applying 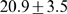 rearrangements (inversions, translocations, duplications, and deletions of size of 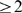) on two different copies that model descendents of the ancestral genome. We ruled out the possibility that a single gene is involved in two rearrangement events. 3) We stored information on positions and types of individual rearrangements. Genes that originate from the same common ancestor and diverged by speciation and duplications are part of the same gene family.

Simulated data represent the only objective way of comparing different softwares. By simulating genome evolution, we know the exact evolutionary history of the synthetic genome. That is why, we are able to unambiguously assign genes to conserved syntenies.

Since CYNTENATOR requires gene similarity information, we assign a bitscore of 1000 as self-similarity score and 500 for orthologous and paralogous proteins corresponding to a gene family. We evaluated the performance of CYNTENATOR, MCMuSec and OrthoCluster on the 20 simulated genome pairs. Program parameters were varied in repeated runs: CYNTENATOR gap and mismatch penalty parameters were set to 0.1, 0.5, 1, 2, 3, 5, 20, the alignment score threshold was set to 1, the minimal alignment length to 2, the maximal number of retained alignments to 1,000 and the gene coverage filter to 4. MCMuSeC was run to report gene clusters, which are shared by at least two genomes. The maxgap value was varied between 1 and 12 and could be interpreted as the number of allowed gene insertions. OrthoCluster was configured to detect all blocks of minimal size two that are conserved in terms of gene order and orientation. The in- and out-mismatch parameters were varied between different runs (1, 2, 5, 10 for both parameters). We constructed correspondance files for OrthoCluster by enumerating all tuples, having a direct BLASTP homology. We defined MCMuSeC homologous groups as all connected components in the BLASTP homology graph.

### Multiple Vertebrate Genome Alignments with CYNTENATOR

We used EnsEMBL database annotations (release 50) for 17 high-coverage genomes to construct multiple gene order alignments. All BLASTP homology scores were retrieved from the EnsEMBL compara database. We used a subtree from the phylogenetic tree by Miller et al. [24] as a guide tree for the progressive alignment (Figure 1). Mismatch and gap parameters were set to 0.3 and the local alignment threshold was set to 2.0 [20]. Other parameters were set as follows: maximal gene coverage to 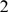 and maximal alignment number to 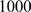.

We modified our parameter choice for the comparison with amniote conserved syntenies of length 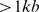 from Larkin et al. (Figure **S3**, [25]). To detect smaller conserved syntenies, we retained maximally 3000 alignments at each ancestral node, increased the gene coverage parameter to 4 and lowered the alignment score threshold to 1.

## Results

### Effect of Parameter Choice and Comparison to Other Tools

#### Gene ortholog recovery

We have previously shown that gene ortholog assignments, as predicted by the EnsEMBL pipeline, are almost fully recovered by our gene order alignments [20]. To this end, we lowered the alignment score threshold such that even single gene pairs were reported (alignment length 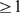). We could show that 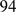
% of all EnsEMBL 1∶1 human-mouse orthologs were correctly recovered. We evaluated the effect of parameter choice on ortholog recovery by computing human-zebrafish gene order alignments using various (mismatch × gap) -penalty combination and apart from that default parameters. Starting with the highest scoring alignments, we greedily extracted one-to-one gene ortholog pairs and compared them to human zebrafish one-to-one orthologs as defined in EnsEMBL release 50. In general, the length of alignments increases with decreasing gap penalty, however also the number of correctly assigned ortholog pairs rises up to 38% of all human-zebrafish EnsEMBL orthologs (Figure **S4**). On the other hand, a variation of the mismatch penalty does not show a strong effect on ortholog assignments and alignment length. In essence, gene order is only retained for the minority of gene ortholog pairs in human-zebrafish comparison.

#### Pairwise comparisons of simulated genomes

**Figure 3:**
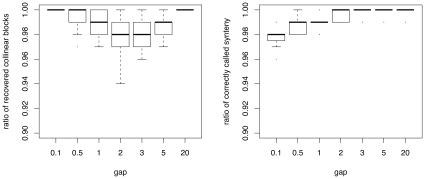
Quality of predicted blocks. We assessed the capacity of CYNTENATOR to detect conserved syntenic blocks under various gap and mismatch combinations using simulated data. Every box corresponds to a fixed gap paramter combined with 7 mismatch parameters on 20 different data sets. We computed the ratio of perfect colinear blocks for which every gene pair was also found in an alignment and the ratio of genes, predicted to be syntenic, that are also located in a simulated blocks.

We also assessed the ability of CYNTENATOR, MCMuSeC and OrthoCluster to detect pairwise conserved syntenic regions. We chose to use simulated data (Figure 2) to provide a “gold standard” as we are able to track all rearrangement events *in silico*. To this end, we used a simplistic approach to simulate genome evolution for a single speciation event. We measured the sensitivity of a method by computing the proportion of simulated blocks with perfect colinearity, which are recovered in a single gene order alignment or identified gene cluster. A simulated block was counted as recovered, if all genes in the block are also found in a single gene order alignment or identified gene cluster. We measure the specificity of the predicted blocks by computing the proportion of genes from all reported alignments or clusters that are also located in simulated blocks. Both performance measures do not consider collinearity. This was done in order to compare methods like MCMuSeC, that do not explicitly test for collinearity. Figure 3 shows that CYNTENATOR predictions are robust to parameter choices over a wide range of different parameter settings. Median performance values are always above 98%. A perfect prediction was obtained, if more conservative parameter settings were applied (gap penalty of 20 and mismatch between 0.5 and 20). More extreme parameter combination will force the alignment either to always introduce a gap or never. Variation of the mismatch parameter does not show a great effect (Figure **S5**). OrthoCluster performed almost perfect under all tested parameter combinations (Figure **S6**), whereas MCMuSeC could never identify all original blocks (Figure **S7**).

#### Multiple comparisons by 17-way gene order alignments

We extended our pairwise analysis to multiple gene order comparisons. We selected 17 high-coverage vertebrate genome assemblies from the EnsEMBL database (release 50) to run multiple gene order comparisons. Table 2 provides an overview on some basic genome assembly parameters. Figure 1 shows the phylogenetic relationship between the 17 vertebrate species [24]. This tree was used to guide the alignment order of CYNTENATOR.

**Table 2:** Genome statistics.

Scientific Name	Name	Seq. Coverage	Size (Gb)	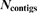	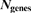
*Homo sapiens*	Human	Fin.	3.2	85	21,529
*Pan troglodytes*	Chimp	6.0 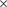	3.3	51	19,830
*Macaca mulatta*	Rhesus	5.1 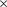	2.9	751	21,906
*Mus musculus*	Mouse	Fin.	2.7	137	23,494
*Rattus norvegicus*	Rat	7.0 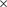	2.7	23	22,504
*Bos taurus*	Cow	7.1 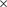	2.7	1,133	21,037
*Canis familiaris*	Dog	7.6 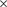	2.5	42	19,306
*Equus cabalus*	Horse	6.8 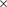	2.4	99	20,323
*Monodelphis domestica*	Opossum	6.5 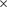	3.6	12	19,472
*Ornitorhynchus anatinus*	Platypus	6.0 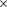	1.2	8,234	17,952
*Gallus gallus*	Chicken	6.6 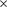	1.1	53	16,737
*Xenopus tropicalis*	Frog	7.9 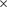	1.2	2,544	18,024
*Tetraodon nigrovirides*	Tetraodon	7.9 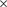	0.4	28	19,603
*Takifugu rubripes*	Fugu	8.5 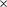	0.3	1,931	18,524
*Gasterosteus aculeatus*	Stickleback	6.0 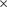	0.4	561	20,788
*Orizya latipes*	Medaka	6.7 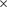	0.8	887	19,687
*Danio rerio*	Zebrafish	6.5 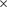	1.4	959	21,323

17 high coverage genome assemblies are included in our comparison. Sequence coverages have been taken from [24]. Genome size, number of contigs and genes have been computed from the EnsEMBL release 50 annotations. The number of contigs for the human genome includes unplaced contigs, haplotype and mitochondrial chromosomes (NT_113917, c6_COX, MT).

Each gene order alignment defines conserved syntenic regions over at least two or more species (CSMs = conserved synteny over multiple species). Our algorithm could identify multiple homologs of one genomic region, which are ranked by their score (Figure **S1**). For example, the human HOXD cluster is homologous to the HOXD and HOXA clusters in chicken. Consequently, the HOXD cluster would be aligned to both loci from chicken by two overlapping local alignments.

We further define a representative syntenic block (RSB) for each set of overlapping CSMs. The RSB is the one that spans the largest genomic regions of all overlapping CSMs. In short, RSBs are non-redundant, maximal representatives for a set of multiple gene order alignments. Table 3 gives an overview of number and sizes of RSBs at the inner nodes in the phylogenetic tree. The number of RSBs is our estimate on the number of conserved syntenic regions in the listed genome comparisons. This number is determined by the process of whole genome evolution as well as the quality of all genome assemblies.

**Table 3:** Overview of conserved synteny in 17 vertebrate genomes.

Comparison	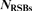	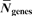	Mean size (Mb)	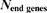
human chimp	32	20,024.0	2,945.1	32
primates	45	19,548.0	2,767.8	31
rodents	87	21,685.0	2,493.0	30
primate rodent	311	18,195.8	2,370.2	53
horse dog	164	18,888.0	2,255.5	76
laurasiatherians	297	17,639.3	2,141.7	120
eutherian mammals	438	15,530.4	1,872.9	77
including opossum	699	13,615.3	1,750.5	73
including platypus	859	6,804.6	934.4	480
amniotes	769	5,930.4	816.9	359
including frog	694	3,774.1	462.1	398
17 vertebrates	287	928.6	75.8	112
5 fish	1,561	7,435.8	184.7	293
stickleback medaka	537	16,125.5	478.3	170
tetraodon fugu	803	15,339.5	255.6	617

Each comparison represents an inner node in the phylogenetic tree (Figure 1). In order to remove redundancy we defined representative syntenic blocks (RSBs) as gene order alignments that have a maximum size among all overlapping alignments that might be due to duplications in one of the lineages. From the set of all RSBs, we computed the mean number of syntenic genes per species and size of the spanned genomic regions. The column ‘
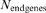
’ gives a rough estimate on how many blocks may be disrupted due to incomplete genome assemblies. It denotes the number of RSBs, in which one of the genes in the alignment is the last gene of a chromosome or contig.

#### Comparison to OrthoCluster and MCMuSeC

Algorithms that are based on set enumerations are not restricted to identifying colinearity. They rather identify genes, which cooccur on the same genomic regions in different species and satisfy additional constraints. This comes at the price of having to explore an exponentially growing search space in the worst case. Previous approach for multiple genome comparison have been shown to perform well on a number of bacterial genomes [13], [14], but they are not specifically designed for vertebrates.

We tested two recent approaches, OrthoCluster and MCMuSeC on their applicability to vertebrate genomes. We tried to repeat the same multiple vertebrate genome comparisons using either OrthoCluster or MCMuSec. None of the two programs could manage this problem size. For example, we applied MCMuSeC on our pairwise comparison of human and mouse with a maxgap parameter of 2. This approach did not finish after one week of computation on an Intel Xeon processor with 2.66GHz. A more elementary difficulty of MCMuSeC stems from the input data. Homologous gene groups are defined as connected components in a gene graph. This way of defining homology could result in “a giant component” and few smaller components. Shared domains between proteins and gene fusions cause this effect.

For OrthoCluster we already noticed a strong increase in running times on the simulated data sets, whereby the running time increased dramatically for higher in-mismatch parameters. When applying OrthoCluster to the human mouse data set, we observed a similar trend as with MCMuSeC. We also noticed that the precomputed correspondence files from OrthoClustDB [26] contain far less homologous gene pairs than the EnsEMBL database (release 50). For example, we found that the OrthoClustDB human-mouse correspondence file contains only 19,309 entries, whereas the EnsEMBL data lists 157,523 homologies. In addition OrthoCluster uses an unfavorable format for correspondence files (enumeration of all homologous tuples required), which would yield a file of more than 100GB for the 17 vertebrate data. In summary, MCMuSeC and OrthoCluster depend on a restrictive preprocessing of homology information. A priori homology assignment by methods like best-reciprocal hits, clusters of orthologous genes (COGs) [27], and the approaches of EnsEMBL [28] and Inparanoid [29] do not consider the genomic context. CYNTENATOR, as well as SYNTENATOR, integrate both signals, gene level similarity and genomic context in an elegant way.

#### Comparison to amniote homologous synteny blocks

Since none of the aforementioned methods worked for our multiple gene order comparison in vertebrates, we compared CYNTENATOR CSMs to a set of multiple vertebrate species syntenic blocks defined by Larkin et al. [25]. These blocks have been constructed using pairwise comparisons of orthologous markers and radiation hybrid maps. In short, this is the only data set we found, which was constructed from gene/marker order alignments. Some of the genomes have not been sequenced with high coverage, for this reason we did not run CYNTENATOR on exactly the same species set. The species set by Larkin et al. encompasses human, chimp, macaque, rat, mouse, pig, cattle, dog, opossum, and chicken data, whereas our data set encompasses the genomes of human, chimp, macaque, rat, mouse, horse, cattle, dog, opossum, and chicken. From this species set we constructed a 10-way amniote multiple alignment and compared the human locations from the resulting CSMs to the human locations of blocks, that were defined by Larkin et al. ([25], Figure **S3**).

The CYNTENATOR CSMs were distributed over 1,399 regions that spanned 1798.8 Mb. 812 blocks from Larkin et al. spanned 1785.2 Mb of which 735 blocks spanning 1477.4 Mb (83%) overlapped with the CYNTENATOR blocks. This corresponds to 77 (10%) regions from Larkin et al. and 548 (39%) of CYNTENATOR region with no overlap in the other data set. Figure **S3** shows the intersection of the two data set on a karyogram. Although there is a substantial overlap between the two data sets, some of the differences might be explained by the fact, that we exclusively used whole genome assemblies whereas Larkin et al. used radiation hybrid maps for cow and pig instead and whole genome assemblies for the remaining species.

#### Genome assembly quality affects gene order alignments

Some genome sequences are distributed over more than 1000 supercontigs or scaffolds. We assessed the impact of this phenomenon on our analyzes by calculating how many alignments might end prematurely because of a contig boundary (Table 3). The platypus genome is the most fragmented genome in our collection. In a multiple gene order alignment of 9 mammalian species and the platypus genome, 480 (56%) of the 859 RSBs are confined by a gene which is located at one end of a contig. This indicates that more than half of the RSBs could potentially be extended or fused if a better platypus genome assembly was available. An example is given of this is shown in Figure **S8**.

If we assume that breakpoints simply arise due to highly fragmented genome assemblies, we could even use our method to build larger genomic scaffolds by merging contigs where end genes are clearly homologous to an adjacent gene pair in a reference species. We leave a careful investigation of this application for future work.

### Functional Analysis of CSMs

As mentioned in the introduction, a number of biological mechanisms is likely to play a role in the retention or breakup of gene orders. In the following analyses, we tested CSMs for an enrichment of experimentally identified enhancer regions [30] and for an enrichment of bidirectional promoters. Regions outside of CSMs were scanned for the abundance of sequence features like transposable elements.

#### P300 bound regions are enriched in conserved syntenic regions

A number of studies have reported correlations between gene expression, function and gene order. Kikuta et al. [8] report evidence for a mechanism, which could maintain long-range conserved synteny across vertebrate genomes. They found conserved chromosomal segments in human-zebrafish comparisons to be spanned by highly conserved non-coding elements, one developmental regulatory ‘target genes’, and phylogenetically and functionally unrelated ‘bystander’ genes. They coined the term genomic regulatory blocks (GRBs) for these regions. The so called ‘bystander’ genes often serve housekeeping functions [31]. The actual evolutionary constraint on the gene order is given by the association of the cis-regulatory elements to their ‘target genes’ [8], [31].

Since the role of highly conserved non-coding regions as enhancers, which are active during embryonic development has already been extensively characterized [7], we used an independent set of experimentally identified enhancers [30] to test for gene order constraints in enhancer regions. These enhancers were identified by massive parallel sequencing of P300 bound regions (ChIP-Seq). The P300 gene encodes an acetyltransferase and transcriptional coactivator which constitutes a general component of enhancer-associated protein complexes and is required for embryonic development [32], [33].

Consequently, this data set has no ascertainment bias for conserved genomic regions. We used this data set to test whether enhancers are enriched in CSMs. We ran a test for enrichment based on random samples at each ancestral node in the mouse lineage of the given phylogeny (see Methods **S1**). Our simulations demonstrate that CSMs are enriched for enhancers in all ancestral nodes (Table **S1**).

#### Bidirectional promoters contribute to the deep conservation of gene pairs

A second regulatory feature, which might constrain gene orders, consists of bidirectional promoters [9]. A selective pressure on gene order could be given in this context, if the expression levels of two neighboring genes are controlled by a common promoter and these expression levels are not free to evolve [34]. We call a gene pair in this configuration, a head-to-head gene pair (H2H). 1,054 head-to-head gene pairs exist in the human genome. We tested whether they are enriched in CSMs, which include the human genome. We observed a significant enrichment of H2H pairs in all CSMs that predate the primate rodent ancestor (Table **S2**, Methods **S1**). To clearly distinguish this observation from P300 binding, we tested all mouse H2H pairs for enrichment of P300 bound regions and did not find a significant enrichment (139 P300 bound regions in H2H pairs in comparison to an expected value 138.4, 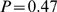). This indicates that the cause for the observed conservation of synteny in H2H pairs is different from the one as described by Kikuta et al [8].

### Recent Evolutionary Breakpoint Regions Exhibit Features of Species-Specific Adaptations

Ohno [35] postulated that only few regions outside of conserved syntenic region are needed for species-specific adaptation processes in evolution. Larkin et al. [25] denote these regions as evolutionary breakpoint regions (EBRs). We performed a Gene Ontology (GO) enrichment analysis for all human genes, which were were outside of CSMs as defined by the human-chimp-macaque gene order comparison (Methods **S1**), assuming that conserved synteny has been lost due to a rearrangement, that introduced an evolutionary breakpoint in this region. We found that immune response related terms like *MHC protein complex* (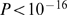) and *NF-kappaB binding* (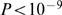), as well as olfactory receptor activity (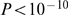) are enriched in such EBRs (Table **S3** and **S4**). Such categories are frequently found in regions that are under positive selection [36] and they were also reported by Larkin et al. [25]. Larkin et al. also found enrichments of structural variants (segmental duplications, copy number variants, and indels), retrotransposed genes and zinc finger genes in EBRs that are shared among multiple species.

Our results show a strong enrichment for *nucleic acid binding* (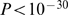) and *zinc ion binding* (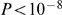) in EBR genes for which synteny was lost at the primate rodent split (Table **S5**). Although the results are less reliable due to the accumulated bias introduced by incomplete genome assemblies, we observed a significant enrichment for the GO term *sensory perception of light stimulus* in EBR genes after the platypus split from the other mammals (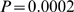) and in EBR genes (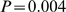) after the split of amniota and amphibia. We also report a mild enrichment for the GO term *sensory perception of mechanical stimulus* in EBR segments (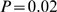), which were formed after the split of Actinopterygii and Sarcopterygii.

#### EBRs are enriched in a variety of transposable elements

Segmental duplications and repetitive elements may contribute to the fragility of genomic regions by increasing the rate of non-allelic homologous recombination [25], [37]. That is why, we tested EBRs at each ancestral split in the human lineage for enrichment of repetitive and transposable elements. We observed that 63 out of 1,083 annotated repeat classes are significantly enriched in EBRs. The strongest enrichment was detected for primate-specific and mammalian-specific LINE-1 elements (L1) in EBRs that originated early in the subtree of mammalian species (Table **S6**, Methods **S1**).

## Discussion

In this work we have extended our previous approach for detecting conserved gene orders [20] to multiple species comparison of dozens of vertebrate genomes. We have recast this problem into a progressive alignment setting by implementing local profile-profile alignments of gene orders. Our new software, CYNTENATOR, computes multiple gene order alignments progressively in a bottom-up approach along a given phylogeny. CYNTENATOR determines the landscape of gene order conservation across distantly related genomes where traditional alignment concepts fail.

We have used the 17-way multiple gene order alignment to define conserved syntenic regions over multiple species (CSMs) and complementary evolutionary breakpoint regions (EBRs). These regions were analyzed for different mechanisms that could preserve or disrupt synteny after species splits.

We showed that regulatory elements such as experimentally identified enhancers [30] are enriched in CSMs and may contribute to the conservation of synteny. We also showed that relative gene order of head-to-head gene pairs is preferentially retained. These genes are often coregulated by means of bidirectional promoters [9].

Common to those two classes of conserved synteny are elements of transcriptional regulation. We just begin to understand what these elements are, how they are distributed and what their target genes are.

On the other hand genes that linked to responses to external stimuli like immune response or sensory perception show elevated levels of sequence variation, segmental duplications and retrotransposition as compared to the genomic average [25]. Evolutionary adaptation takes place in evolutionary breakpoint regions, where certain gene categories and specific repetitive elements are significantly enriched. Herein, we reported a clear pattern of gene enrichment for the human lineage: Genes related to chemosensation and immunity preferentially reside in primate breakpoint regions. Genes related to nucleic acid binding and nucleic acid metabolism reside in EBRs of the primate-rodent split.

Central to the CYNTENATOR algorithm is the progressive alignment methodology, which scales to dozens of vertebrate genomes. CYNTENATOR implements a phylogenetic scoring function, which weights gene pairs according to their position in the phylogenetic tree.

In a comparison of CYNTENATOR to other existing methods, we found that the definition of homology is an essential aspect in terms of accuracy and speed. Programs like OrthoCluster and MCMuSeC require a restrictive preprocessing of homology data, which could result in incorrect synteny predictions. CYNTENATOR uses all-against-all gene similarity scores as input and does not require a restrictive homology assignment. It performs as good as state-of-the art programs in pairwise comparisons on simulated data sets and is the only software that could be directly applied to 17 vertebrate genomes.

In summary, CYNTENATOR represents a flexible tool to study chromosome rearrangements and genome evolution.

## Supporting Information

Figure S1 AB denotes a genomic region with genes A and B. (A) After duplication and speciation, each successor species has two copies of this cluster. (B) Similarities in terms of alignment scores between gene clusters are shown as a bipartite homology graph. (C) As long as the top ranking alignment is correctly assigned, the unique filter will discard wrong assignments (assignments that do not correspond to the more recent evolutionary event, e.g., speciation). If only binary homology data is used, no decision can be made.
(0.01 MB PDF)

Figure S2 Comparison of gene coverage parameters from human mouse alignments. CYNTENATOR was run on the human and mouse data with mismatch and gap penalty 0.3 and a minimum alignment score threshold of 2. The alignment number filter was set to 10000. The y-axis denotes the number of aligned gene pairs for varying gene coverage parameters.
(0.00 MB PDF)

Figure S3 Comparison of amniote CSMs. We built an alignment of ten amniote species (human, chimp, macaque, mouse, rat, cow, dog, horse, opossum, and chicken) and compared the human locations from the resulting CSMs to the corresponding locations from msHSBs from Larkin et al. Although some msHSBs were identified by only one method (e.g., lower arm of chromosome 4), which may be due to different assembly qualities and species sets, both sets largely agree.
(0.48 MB PDF)

Figure S4 Exploration of parameter space. For various combinations of mismatch and gap penalty, we computed human zebrafish gene order alignment and greedily extracted one-to-one pairs from set of local alignment, ordered by decreasing score. We counted which percentage of the 8,001 human zebrafish one-to-one orthologs from Ensembl release 50 could be recovered. The right graph shows the total length of the alignments in genes times 1,000. Decreasing the gap penalty increases the length of the alignments; however, also, more “true” one-to-one relationships could be recovered as highest scoring pairs. This indicates that lowering of this parameter does not correlate with the assignment of false homologies. Variation of mismatch parameter does not have a large effect on both measures.
(0.10 MB PDF)

Figure S5 CYNTENATOR performance for various mismatch parameter settings.
(0.01 MB PDF)

Figure S6 OrthoCluster performance for various parameter settings.
(0.01 MB PDF)

Figure S7 MCMuSeC performance for various parameter settings.
(0.01 MB PDF)

Figure S8 Predicting Ultracontig links in the platypus assembly. A UCSC Genome Browser screenshot is shown, in which two adjacent human-platypus CYNTENATOR alignments are bounded by the end of platypus Ultracontigs 483 and 542; human-platypus net alignments are shown on the lower track. Between the two regions platypus Contig 3692 is located, containing the *Rragd* gene. Assuming that synteny is preserved in this region, Ultracontigs 483, Contig 3692, and Ultracontig 542 might be linked in the platypus assembly.
(0.04 MB PDF)

Methods S1 Detailed description of the P300 peak, head-to-head pair, gene ontology term, and transposable element enrichment analysis of CSMs and evolutionary breakpoint regions.
(0.04 MB PDF)

Table S1 Enrichment P300 bound regions in mouse syntenic blocks. We used mouse enhancer regions, experimentally identified by ChIP-seq of enhancer protein P300 from Visel et al., to test for enrichment in conserved syntenic blocks. We determined p values by repeatedly selecting an equal number of random genomic location of the same length and testing for overlap with the P300 bound regions.
(0.03 MB PDF)

Table S2 Enrichment of head-to-head (H2H) pairs in CSMs. 1,054 (5%) of 21,444 neighboring gene pairs in humans fall under the H2H category (see Methods **S1**). With the exception of the 17 vertebrate blocks, we observed a significant enrichment of H2H pairs in all multiple species syntenic blocks predating the human rodent split. Although the 17 vertebrates shows the highest enrichment in H2H pairs, this was not found to be statistically by the Fisher's exact test with Bonferroni correction.
(0.03 MB PDF)

Table S3 Gene ontology analysis of human genes for which synteny was last after the human-chimp split.
(0.03 MB PDF)

Table S4 Gene ontology analysis of human genes for which synteny was last after the human-chimp vs. macaque split.
(0.03 MB PDF)

Table S5 Gene ontology analysis of human genes for which synteny was lost after the primate rodent split.
(0.03 MB PDF)

Table S6 We evaluated evolutionary breakpoint regions (EBRs) following the human path in the phylogenetic tree by counting occurrences of transposable elements in regions for which synteny was lost after a speciation event. At each node (e.g., primate rodent), node-specific EBR regions from humans were extracted and analyzed. All significantly enriched repetitive elements are marked with a cross (comparison vs. random regions, P<0.001).
(0.02 MB PDF)
