# Supplementary material for: CYNTENATOR: Progressive Gene Order Alignment of 17 Vertebrate Genomes
Source: PLoS One. 2010 Jan 28;5(1):e8861. doi: 10.1371/journal.pone.0008861 (PMC2812507; doi:10.1371/journal.pone.0008861)
Supplement: Figure S1 — AB denotes a genomic region with genes A and B. (A) After duplication and speciation, each successor species has two copies of this cluster. (B) Similarities in terms of alignment scores between gene clusters are shown as a bipartite homology graph. (C) As long as the top ranking alignment is correctly assigned, the unique filter will discard wrong assignments (assignments that do not correspond to the more recent evolutionary event, e.g., speciation). If only binary homology data is used, no decision can be made. (0.01 MB PDF) [file pone.0008861.s001.pdf]

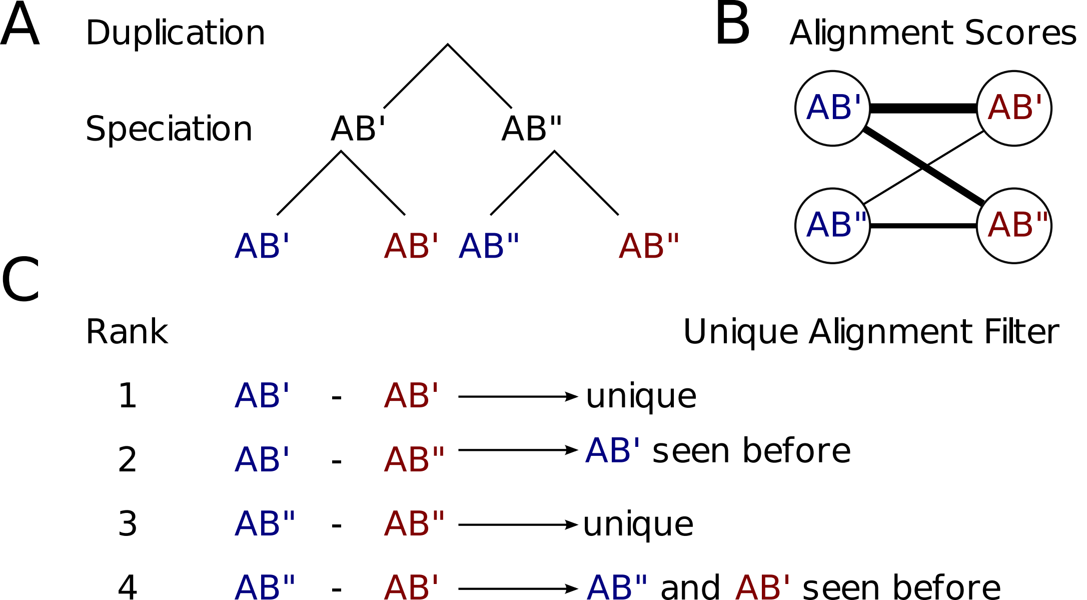
A
Duplication
Speciation
AB'
AB"
AB'
AB'
AB"
AB"
B
Alignment Scores
AB'
AB'
AB"
AB"
C
Rank
Unique Alignment Filter
1 AB' - AB' unique
2 AB' - AB" AB' seen before
3 AB" - AB" unique
4 AB" - AB' AB" and AB' seen before
