# Supplementary figures and images for: CYNTENATOR: Progressive Gene Order Alignment of 17 Vertebrate Genomes
Source: PLoS One. 2010 Jan 28;5(1):e8861. doi: 10.1371/journal.pone.0008861 (PMC2812507; doi:10.1371/journal.pone.0008861)

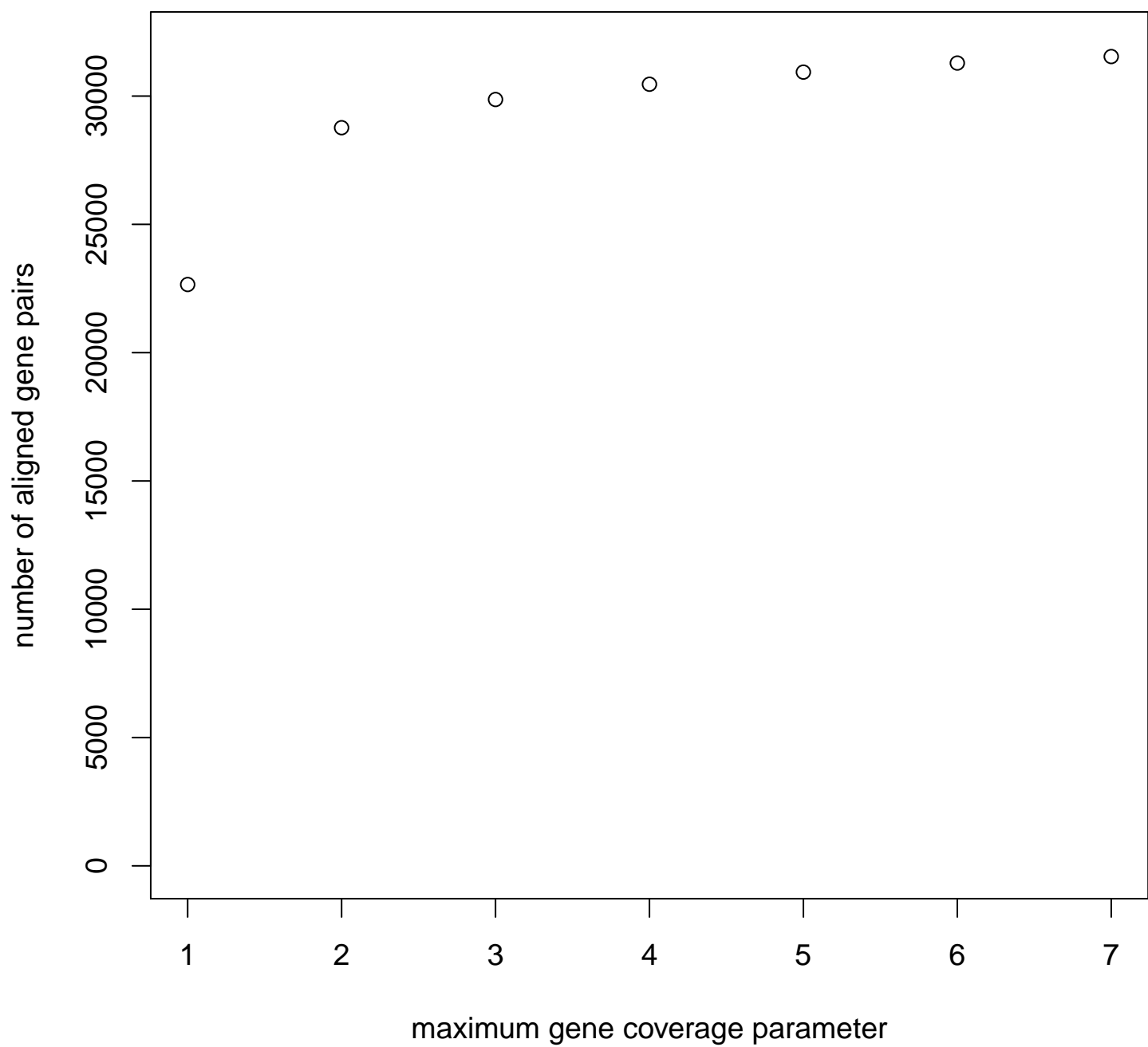

Supplement: Figure S2 — Comparison of gene coverage parameters from human mouse alignments. CYNTENATOR was run on the human and mouse data with mismatch and gap penalty 0.3 and a minimum alignment score threshold of 2. The alignment number filter was set to 10000. The y-axis denotes the number of aligned gene pairs for varying gene coverage parameters. (0.00 MB PDF) [file pone.0008861.s002.pdf]

■ Larkin et al.  
■ CYNTENATOR

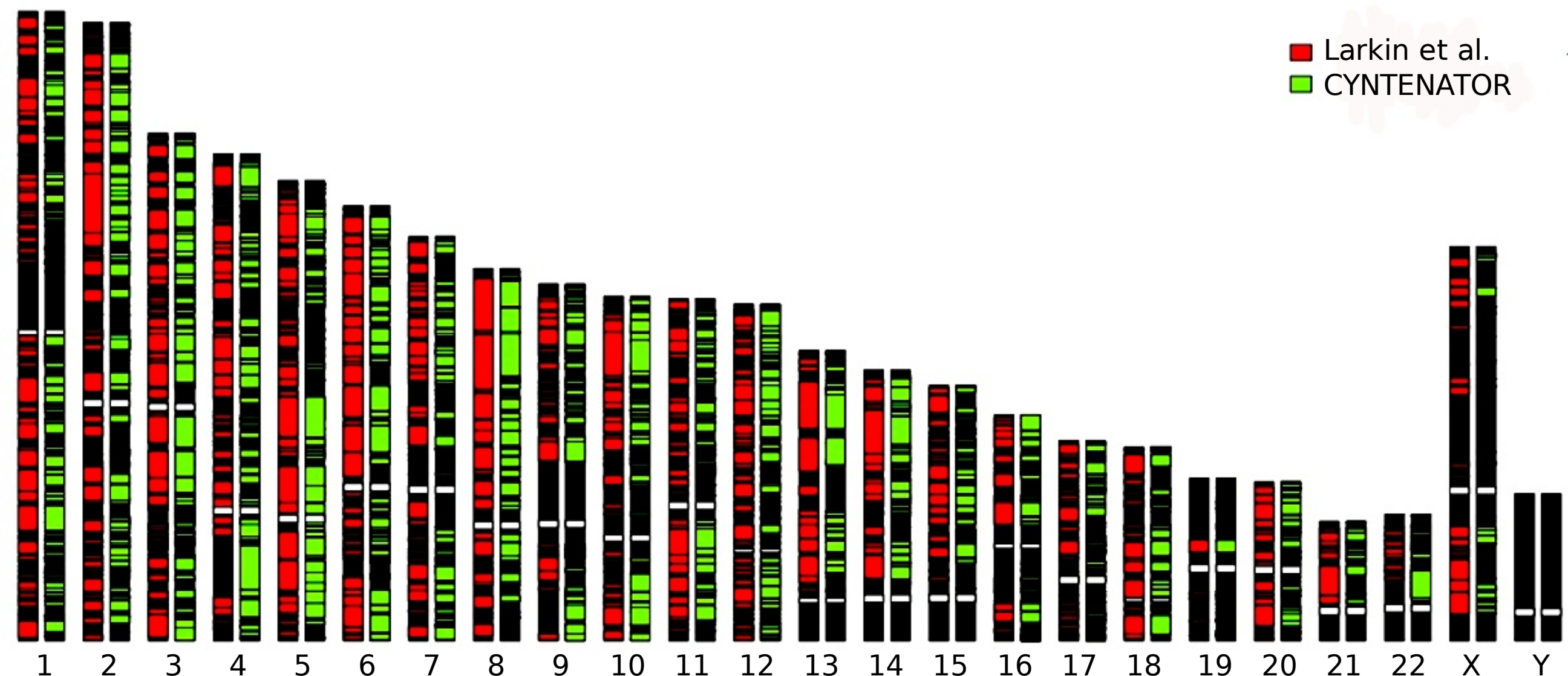

Supplement: Figure S3 — Comparison of amniote CSMs. We built an alignment of ten amniote species (human, chimp, macaque, mouse, rat, cow, dog, horse, opossum, and chicken) and compared the human locations from the resulting CSMs to the corresponding locations from msHSBs from Larkin et al. Although some msHSBs were identified by only one method (e.g., lower arm of chromosome 4), which may be due to different assembly qualities and species sets, both sets largely agree. (0.48 MB PDF) [file pone.0008861.s003.pdf]

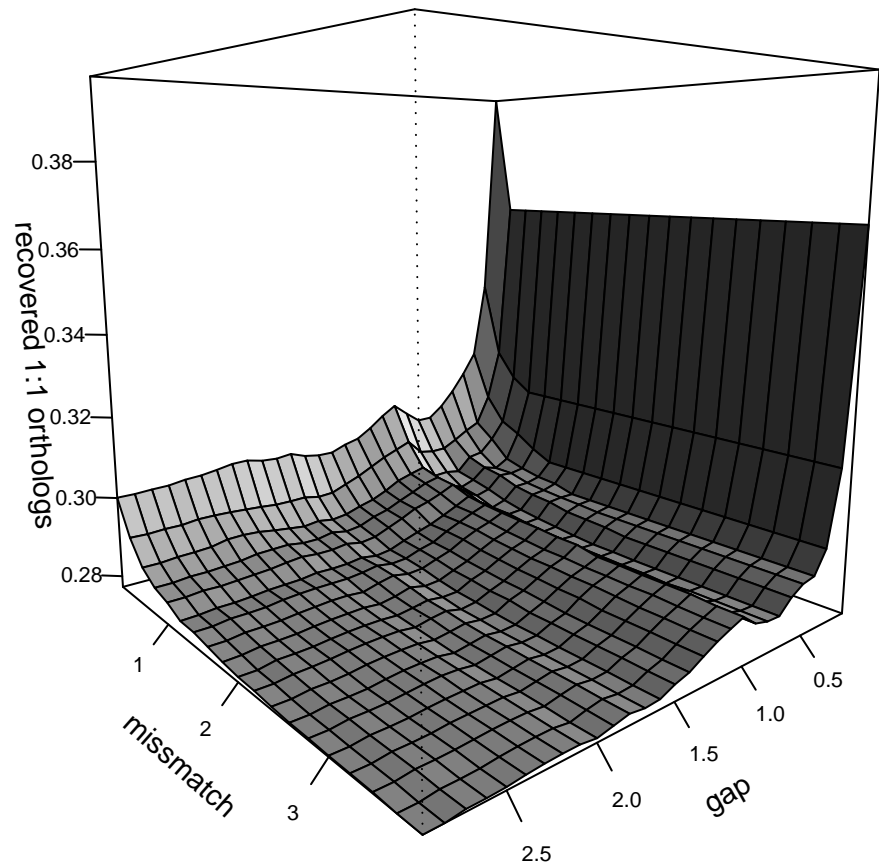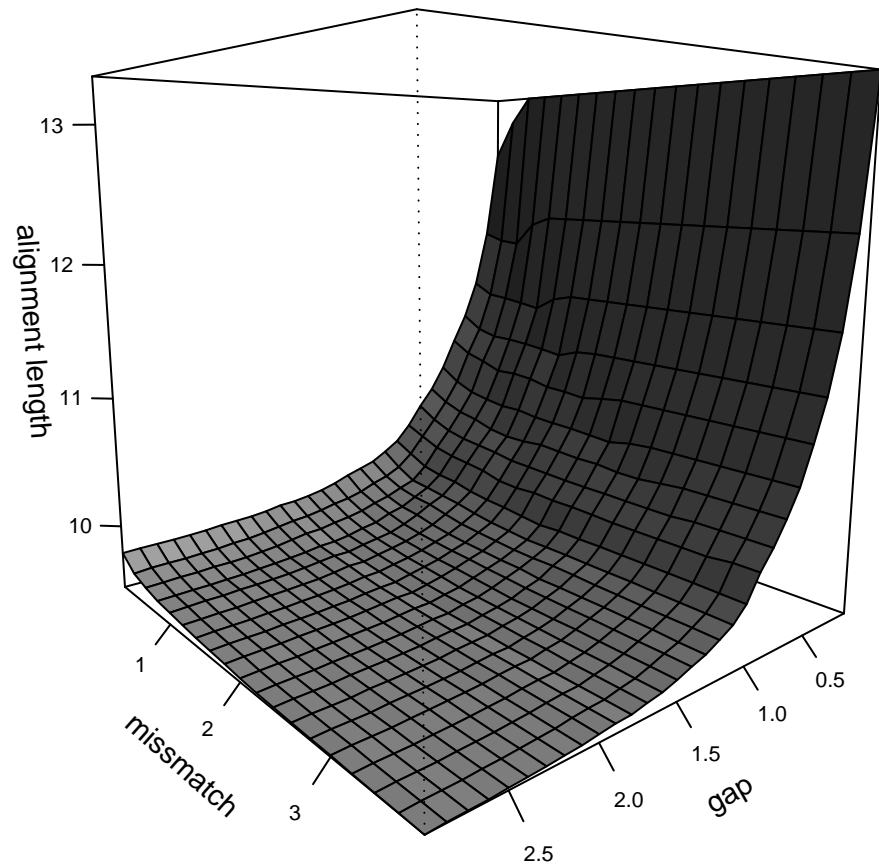

Supplement: Figure S4 — Exploration of parameter space. For various combinations of mismatch and gap penalty, we computed human zebrafish gene order alignment and greedily extracted one-to-one pairs from set of local alignment, ordered by decreasing score. We counted which percentage of the 8,001 human zebrafish one-to-one orthologs from Ensembl release 50 could be recovered. The right graph shows the total length of the alignments in genes times 1,000. Decreasing the gap penalty increases the length of the alignments; however, also, more “true” one-to-one relationships could be recovered as highest scoring pairs. This indicates that lowering of this parameter does not correlate with the assignment of false homologies. Variation of mismatch parameter does not have a large effect on both measures. (0.10 MB PDF) [file pone.0008861.s004.pdf]

ratio of recovered collinear blocks

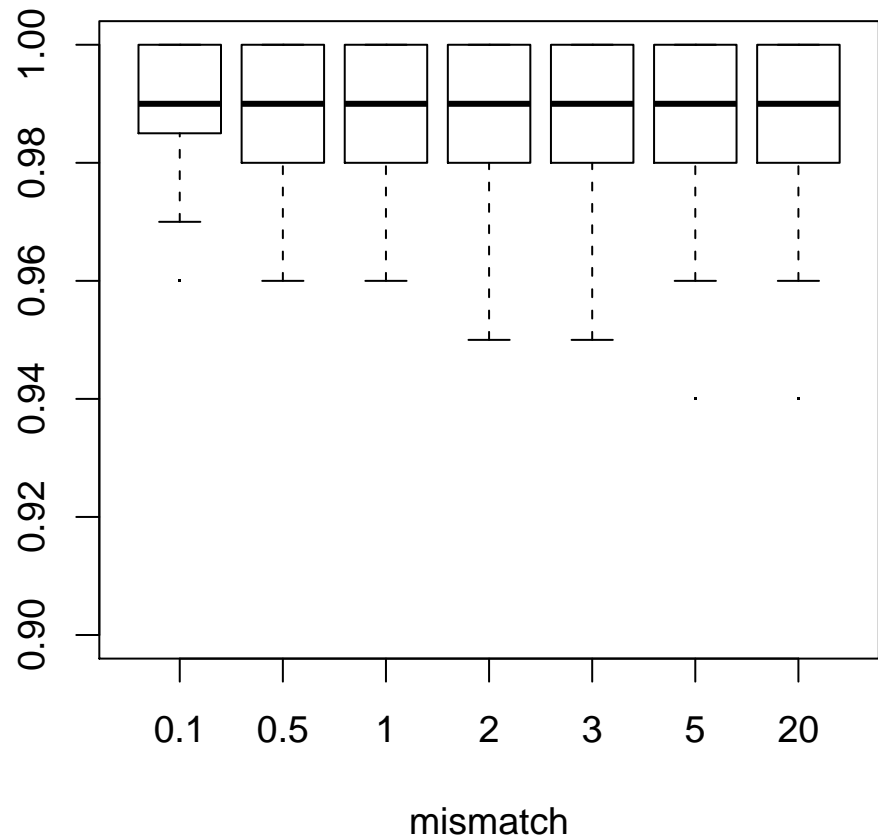

ratio of correctly called synteny

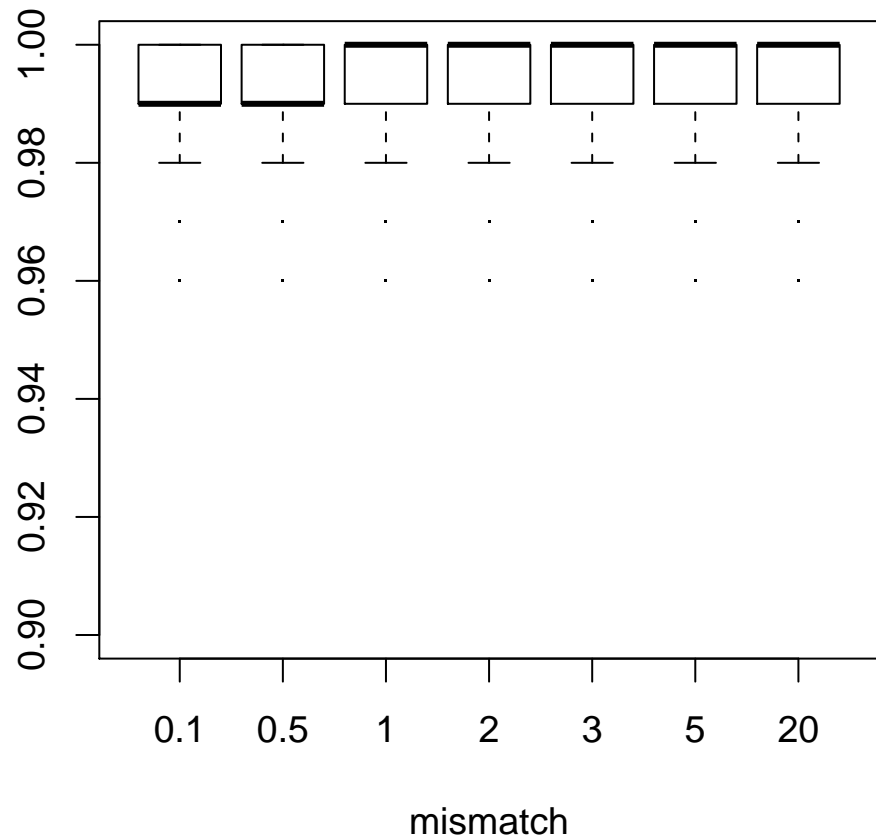

Supplement: Figure S5 — CYNTENATOR performance for various mismatch parameter settings. (0.01 MB PDF) [file pone.0008861.s005.pdf]

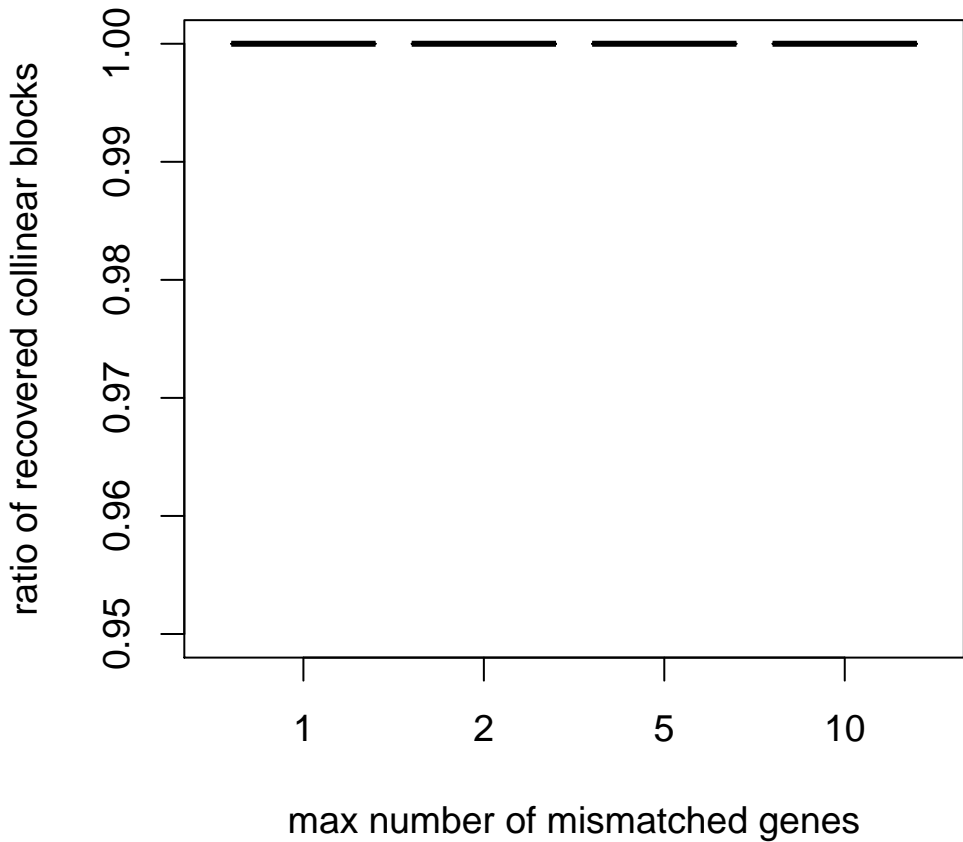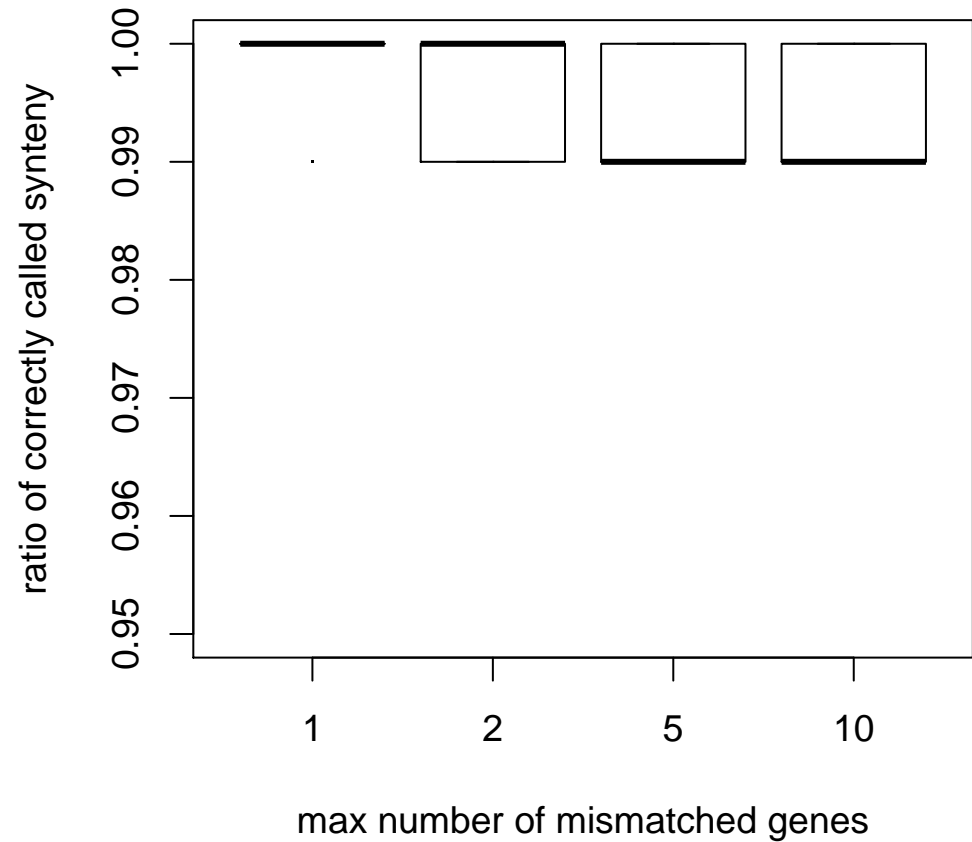

Supplement: Figure S6 — OrthoCluster performance for various parameter settings. (0.01 MB PDF) [file pone.0008861.s006.pdf]

ratio of recovered collinear blocks

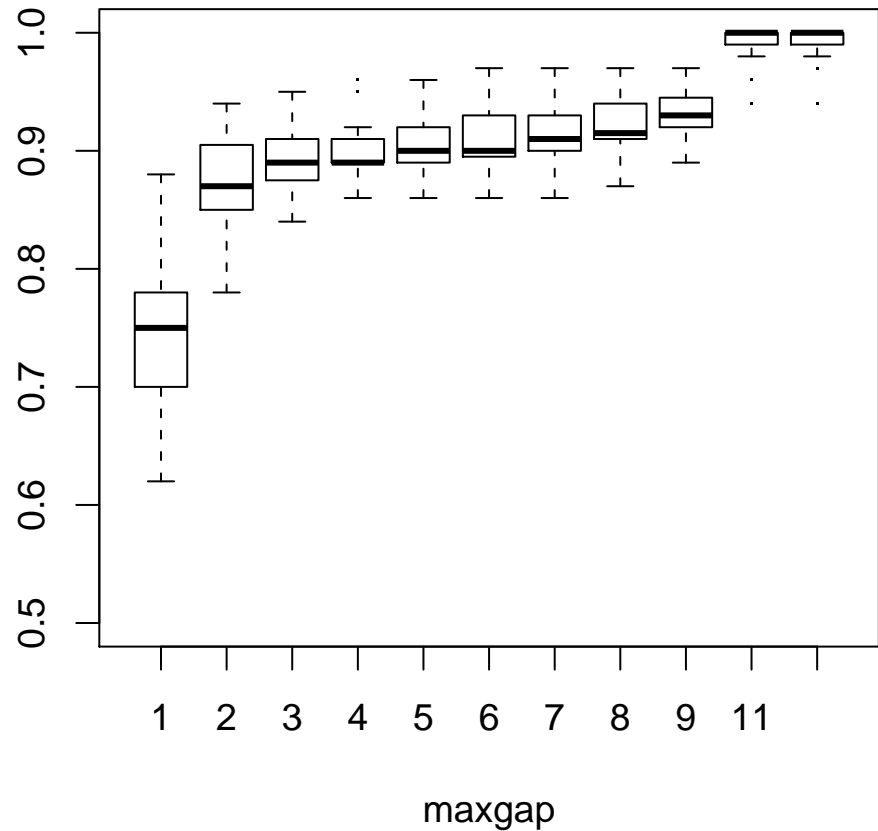

ratio of correctly called synteny

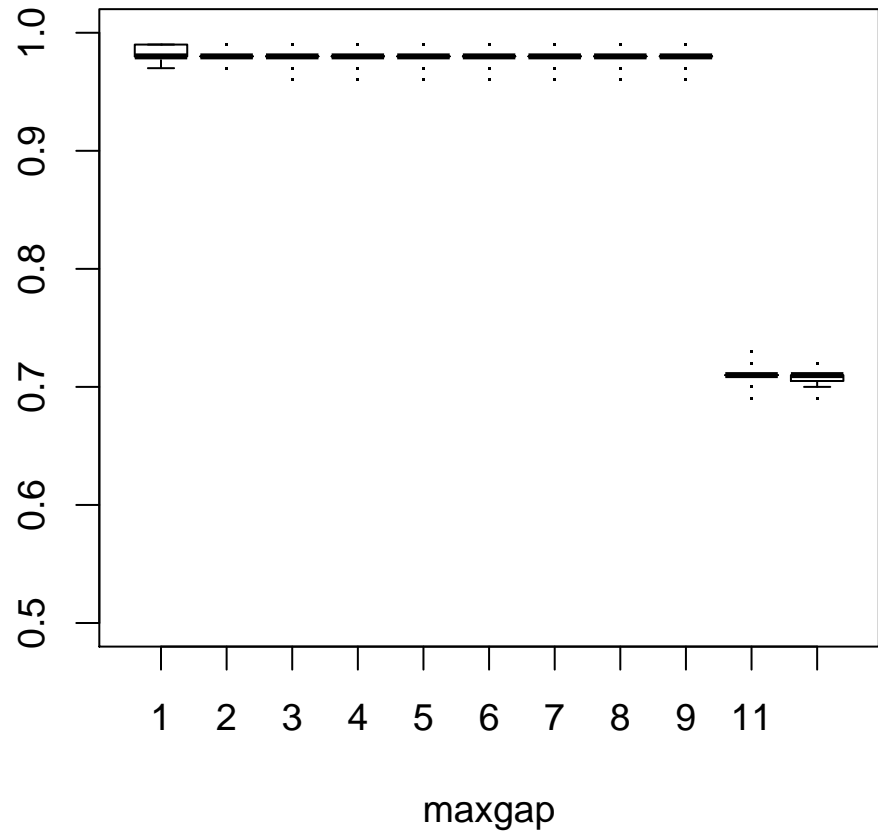

Supplement: Figure S7 — MCMuSeC performance for various parameter settings. (0.01 MB PDF) [file pone.0008861.s007.pdf]

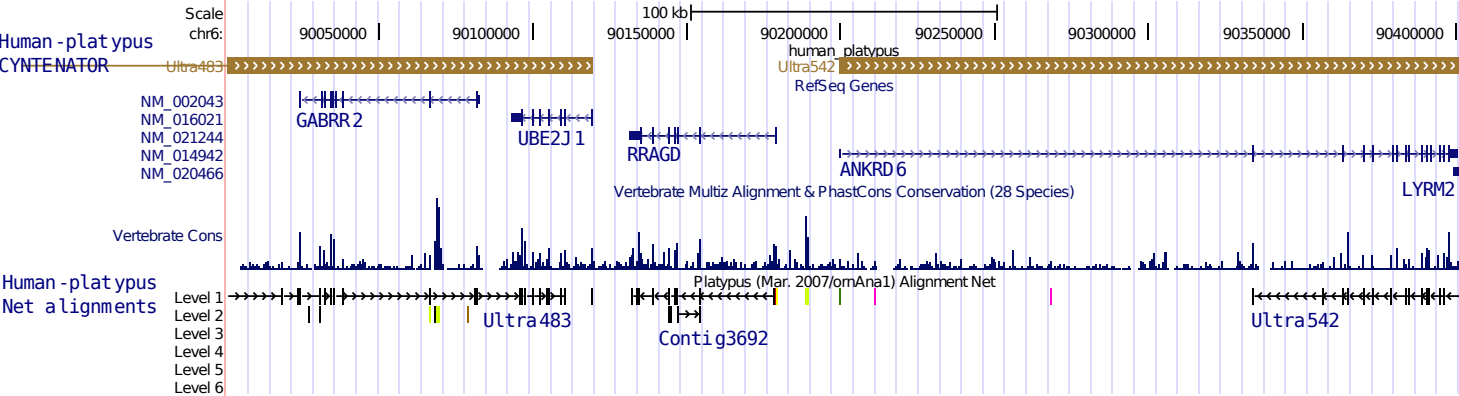

Supplement: Figure S8 — Predicting Ultracontig links in the platypus assembly. A UCSC Genome Browser screenshot is shown, in which two adjacent human-platypus CYNTENATOR alignments are bounded by the end of platypus Ultracontigs 483 and 542; human-platypus net alignments are shown on the lower track. Between the two regions platypus Contig 3692 is located, containing the Rragd gene. Assuming that synteny is preserved in this region, Ultracontigs 483, Contig 3692, and Ultracontig 542 might be linked in the platypus assembly. (0.04 MB PDF) [file pone.0008861.s008.pdf]
