# Supplementary material for: CYNTENATOR: Progressive Gene Order Alignment of 17 Vertebrate Genomes
Source: PLoS One. 2010 Jan 28;5(1):e8861. doi: 10.1371/journal.pone.0008861 (PMC2812507; doi:10.1371/journal.pone.0008861)
Supplement: Methods S1 — Detailed description of the P300 peak, head-to-head pair, gene ontology term, and transposable element enrichment analysis of CSMs and evolutionary breakpoint regions. (0.04 MB PDF) [file pone.0008861.s009.pdf]

Supplementary Methods S1

P300 Enrichment

We downloaded a set of 5119 P300 ChIP-seq peaks from embryonal mouse forebrain, midbrain and limb tissue [1]. We extracted syntenic blocks from the CYNTENATOR alignments using the Ensembl release 50 gene annotations and counted the number of P300 bound regions falling into syntenic blocks at each node including mouse. We calculated p-values by randomly selecting an equal amount of genomic locations with the same length as the syntenic blocks and then counting the P300 bound region in the randomly chosen blocks. This was repeated 1000 times and p-values were calculated as the number of random sets which show equal or higher number of P300 bound regions (Table **S1**).

Head-to-Head Pair Enrichment

We extracted from the Ensembl release 50 *Homo Sapiens* annotation a set of 1054 head-to-head (H2H) pairs that were defined as pairs of two neighboring genes, where the first gene is located on the Crick strand and the second on the Watson strand. Additionally we restricted the distance between their annotated transcription start sites to be $< 1kb$. We extracted pairs from the CYNTENATOR gene order alignments and determined the overlap with the complete set of head-to-head pairs. We calculated p-values using a Fisher's exact test with Bonferroni correction (Table **S2**).

Gene Ontology (GO)

We examined sets of genes, for which collinearity is lost at a certain point in evolution for enrichment of Gene Ontology (GO) terms. We first took all genes that could not be found in any human-chimp gene order alignment and tested this set for GO enrichment using the Ontologizer program [2] (Table **S3**). We then extracted all genes that are not conserved in the primate gene order alignment and excluded the previously analyzed human-chimp-non-syntenic genes in order to test for GO enrichment of genes for which the synteny was disrupted after the rhesus and human-chimp split (Table **S4**). This was repeated at each further node in the phylogenetic tree including human.

Enrichment in Transposable Elements

We evaluated evolutionary breakpoint regions following the human bottom-up-path in the phylogenetic tree by extracting the complementary human genomic regions (EBRs) of the CSMs. We excluded EBRs at inner nodes if they overlapped with EBRs that were already identified at a child node.

We downloaded nested repeat annotation for the human genome (hg18) from the UCSC database [3]. We then counted the number of occurrences for each class of repetitive elements and compared those numbers to randomly chosen genomic regions of the same size distribution. We considered an element as significantly enriched if no random set showed an equal or higher number of occurrences than the observed set in 1000 repetitions.

References

[1] Axel Visel, Matthew J Blow, Zirong Li, Tao Zhang, Jennifer A Akiyama, Amy Holt, Ingrid Plajzer-Frick, Malak Shoukry, Crystal Wright, Feng Chen, Veena Afzal, Bing Ren, Edward M Rubin, and Len A Pennacchio. Chip-seq accurately predicts tissue-specific activity of enhancers. *Nature*, 457(7231):854–858, Feb 2009.

[2] Sebastian Bauer, Steffen Grossmann, Martin Vingron, and Peter N Robinson. Ontologizer 2.0–a multifunctional tool for GO term enrichment analysis and data exploration. *Bioinformatics*, 24(14):1650–1651, Jul 2008.

[3] R. M. Kuhn, D. Karolchik, A. S. Zweig, T. Wang, K. E. Smith, K. R. Rosenbloom, B. Rhead, B. J. Raney, A. Pohl, M. Pheasant, L. Meyer, F. Hsu, A. S. Hinrichs, R. A. Harte, B. Giardine, P. Fujita, M. Diekhans, T. Dreszer, H. Clawson, G. P. Barber, D. Haussler, and W. J. Kent. The ucsc genome browser database: update 2009. *Nucleic Acids Res*, 37(Database issue):D755–D761, Jan 2009.
