# Supplementary material for: CYNTENATOR: Progressive Gene Order Alignment of 17 Vertebrate Genomes
Source: PLoS One. 2010 Jan 28;5(1):e8861. doi: 10.1371/journal.pone.0008861 (PMC2812507; doi:10.1371/journal.pone.0008861)
Supplement: Table S1 — Enrichment P300 bound regions in mouse syntenic blocks. We used mouse enhancer regions, experimentally identified by ChIP-seq of enhancer protein P300 from Visel et al., to test for enrichment in conserved syntenic blocks. We determined p values by repeatedly selecting an equal number of random genomic location of the same length and testing for overlap with the P300 bound regions. (0.03 MB PDF) [file pone.0008861.s010.pdf]

**CSMs**	$\boldsymbol{N}_{\textbf{P300}}$	$\boldsymbol{N}_{\textbf{expected}}$	***p*-value**
rat	5083	3207.8	$< 10^{-3}$
primates-rodents	4852	3958.8	$< 10^{-3}$
eutheraian mammals	4306	3408.2	$< 10^{-3}$
mammals	3926	3134.9	$< 10^{-3}$
including platypus	2033	1672.0	$< 10^{-3}$
amniotes	1888	1555.6	$< 10^{-3}$
including frog	1223	938.1	$< 10^{-3}$
17 vertebrates	308	205.2	$< 10^{-3}$
