# Supplementary material for: CYNTENATOR: Progressive Gene Order Alignment of 17 Vertebrate Genomes
Source: PLoS One. 2010 Jan 28;5(1):e8861. doi: 10.1371/journal.pone.0008861 (PMC2812507; doi:10.1371/journal.pone.0008861)
Supplement: Table S2 — Enrichment of head-to-head (H2H) pairs in CSMs. 1,054 (5%) of 21,444 neighboring gene pairs in humans fall under the H2H category (see Methods S1 ). With the exception of the 17 vertebrate blocks, we observed a significant enrichment of H2H pairs in all multiple species syntenic blocks predating the human rodent split. Although the 17 vertebrates shows the highest enrichment in H2H pairs, this was not found to be statistically by the Fisher's exact test with Bonferroni correction. (0.03 MB PDF) [file pone.0008861.s011.pdf]

**CSMs**	$N_{\mathbf{H2H}}$	$N_{\mathbf{Total}}$	**Enrichment**	***p-value***
human chimp	1,017	20,938	0.99	1
primates	1,014	20,639	1.00	1
primates-rodents	904	15,934	1.15	0.02
eutherian mammals	821	13,832	1.20	0.0008
including opossum	827	13,987	1.21	0.001
including platypus	287	5,471	1.26	0.04
amniotes	296	4,746	1.27	0.005
including frog	166	2,564	1.31	0.02
17 vertebrates	27	405	1.36	1
