# Supplementary material for: CYNTENATOR: Progressive Gene Order Alignment of 17 Vertebrate Genomes
Source: PLoS One. 2010 Jan 28;5(1):e8861. doi: 10.1371/journal.pone.0008861 (PMC2812507; doi:10.1371/journal.pone.0008861)
Supplement: Table S3 — Gene ontology analysis of human genes for which synteny was last after the human-chimp split. (0.03 MB PDF) [file pone.0008861.s012.pdf]

Term	*p*-value	Description
GO:0042611	$< 10^{-16}$	MHC protein complex
GO:0019882	$< 10^{-14}$	antigen processing and presentation
GO:0060089	$< 10^{-13}$	molecular transducer activity
GO:0007606	$< 10^{-12}$	sensory perception of chemical stimulus
GO:0007186	$< 10^{-11}$	G-protein coupled receptor protein signaling pathway
GO:0050896	$< 10^{-11}$	response to stimulus
GO:0004984	$< 10^{-10}$	olfactory receptor activity
GO:0051059	$< 10^{-9}$	NF-kappaB binding
GO:0003008	$< 10^{-8}$	system process
GO:0051789	$< 10^{-8}$	response to protein stimulus
GO:0004872	$< 10^{-7}$	receptor activity
GO:0007166	$< 10^{-7}$	cell surface receptor linked signal transduction
GO:0032395	$< 10^{-7}$	MHC class II receptor activity
GO:0045111	$< 10^{-6}$	intermediate filament cytoskeleton
GO:0005886	$< 10^{-6}$	plasma membrane
GO:0042824	$< 10^{-5}$	MHC class I peptide loading complex
GO:0050890	$< 10^{-5}$	cognition
GO:0004888	$< 10^{-5}$	transmembrane receptor activity
GO:0042287	$< 10^{-5}$	MHC protein binding
GO:0002376	$< 10^{-5}$	immune system process
GO:0050877	0.0002	neurological system process
GO:0007154	0.0002	cell communication
GO:0006952	0.0002	defense response
GO:0044425	0.0004	membrane part
GO:0005634	0.0007	nucleus
GO:0006323	0.0008	DNA packaging
GO:0050785	0.0008	advanced glycation end-product receptor activity
GO:0004930	0.0009	G-protein coupled receptor activity
GO:0006955	0.0012	immune response
GO:0003708	0.0019	retinoic acid receptor activity
GO:0016853	0.0021	isomerase activity
GO:0043933	0.0022	macromolecular complex subunit organization
GO:0031224	0.0023	intrinsic to membrane
GO:0002504	0.0023	antigen processing and presentation of peptide or polysaccharide antigen via MHC class II
GO:0009991	0.0023	response to extracellular stimulus
GO:0043087	0.0023	regulation of GTPase activity
GO:0030545	0.0024	receptor regulator activity
GO:0015197	0.0026	peptide transporter activity
GO:0032196	0.0028	transposition
GO:0008134	0.0028	transcription factor binding
GO:0032813	0.0030	tumor necrosis factor receptor superfamily binding
GO:0043565	0.0033	sequence-specific DNA binding
GO:0042613	0.0034	MHC class II protein complex
GO:0015665	0.0036	alcohol transmembrane transporter activity
GO:0007608	0.0036	sensory perception of smell
GO:0046977	0.0038	TAP binding
GO:0003676	0.0043	nucleic acid binding
GO:0007600	0.0049	sensory perception
GO:0048871	0.0056	multicellular organismal homeostasis
GO:0006414	0.0059	translational elongation
GO:0019865	0.0070	immunoglobulin binding
...		
