# Supplementary material for: CYNTENATOR: Progressive Gene Order Alignment of 17 Vertebrate Genomes
Source: PLoS One. 2010 Jan 28;5(1):e8861. doi: 10.1371/journal.pone.0008861 (PMC2812507; doi:10.1371/journal.pone.0008861)
Supplement: Table S4 — Gene ontology analysis of human genes for which synteny was last after the human-chimp vs. macaque split. (0.03 MB PDF) [file pone.0008861.s013.pdf]

Term	*p*-value	Description
GO:0004984	$< 10^{-7}$	olfactory receptor activity
GO:0007606	$< 10^{-5}$	sensory perception of chemical stimulus
GO:0045111	0.0003	intermediate filament cytoskeleton
GO:0010467	0.0005	gene expression
GO:0003676	0.0016	nucleic acid binding
GO:0007186	0.0193	G-protein coupled receptor protein signaling pathway
