# Supplementary material for: CYNTENATOR: Progressive Gene Order Alignment of 17 Vertebrate Genomes
Source: PLoS One. 2010 Jan 28;5(1):e8861. doi: 10.1371/journal.pone.0008861 (PMC2812507; doi:10.1371/journal.pone.0008861)
Supplement: Table S5 — Gene ontology analysis of human genes for which synteny was lost after the primate rodent split. (0.03 MB PDF) [file pone.0008861.s014.pdf]

Term	*p*-value	Description
GO:0003676	$< 10^{-30}$	nucleic acid binding
GO:0006139	$< 10^{-23}$	nucleobase, nucleoside, nucleotide and nucleic acid metabolic process
GO:0043167	$< 10^{-18}$	ion binding
GO:0010467	$< 10^{-15}$	gene expression
GO:0009058	$< 10^{-15}$	biosynthetic process
GO:0046914	$< 10^{-12}$	transition metal ion binding
GO:0019222	$< 10^{-11}$	regulation of metabolic process
GO:0003677	$< 10^{-11}$	DNA binding
GO:0005634	$< 10^{-9}$	nucleus
GO:0008270	$< 10^{-8}$	zinc ion binding
GO:0016070	$< 10^{-7}$	RNA metabolic process
GO:0043169	$< 10^{-6}$	cation binding
GO:0065007	$< 10^{-6}$	biological regulation
GO:0007606	$< 10^{-5}$	GO:0007606 sensory perception of chemical stimulus
GO:0007186	0.0006	G-protein coupled receptor protein signaling pathway
GO:0050890	0.0020	cognition
GO:0004872	0.0041	receptor activity
GO:0009410	0.0054	response to xenobiotic stimulus
GO:0006952	0.0168	defense response
GO:0043170	0.0364	macromolecule metabolic process
