# Supplementary material for: CYNTENATOR: Progressive Gene Order Alignment of 17 Vertebrate Genomes
Source: PLoS One. 2010 Jan 28;5(1):e8861. doi: 10.1371/journal.pone.0008861 (PMC2812507; doi:10.1371/journal.pone.0008861)
Supplement: Table S6 — We evaluated evolutionary breakpoint regions (EBRs) following the human path in the phylogenetic tree by counting occurrences of transposable elements in regions for which synteny was lost after a speciation event. At each node (e.g., primate rodent), node-specific EBR regions from humans were extracted and analyzed. All significantly enriched repetitive elements are marked with a cross (comparison vs. random regions, P<0.001). (0.02 MB PDF) [file pone.0008861.s015.pdf]

Repeat	human	primate	rodents	eutherian mammals	mammals	platypus	amniote	frog	all
(CA)n						×	×	×	×
(CAAT)n	×								
(TC)n	×								
(TCCCC)n	×								
(TG)n						×			
ALR/Alpha			×	×	×				
AluY						×			
C-rich				×	×				
ERVL-B4				×	×				
HAL1b								×	
HERVK			×	×					
HERVL74				×	×				
HERVS71			×						
L1M1				×	×				
L1M2				×	×				
L1M3				×	×				
L1M3c				×	×				
L1M4					×				
L1M4b					×				
L1M4c				×	×				
L1M5						×		×	
L1MA6				×	×				
L1MA7					×				
L1MA8					×				
L1MA9				×	×		×	×	
L1MCa					×				
L1MCb				×	×				
L1MDb				×					
L1ME1									×
L1ME2									×
L1ME3A									×
L1P4				×	×				
L1PA10				×	×				
L1PA13				×	×				
L1PA15					×				
L1PA16					×				
L1PA4					×				
L1PA8				×	×				
L1PB3					×				
L1PREC2				×					
LOR1-int				×					
LOR1a					×				
LOR1b-int				×					
LTR2C		×		×	×				
LTR35		×	×		×				
LTR41						×			
LTR5A		×							
LTR60	×	×	×						
MER112									×
MER41C	×	×							
MER4A1		×	×	×	×				
MER54A			×						
MER65D	×	×							
MER67C					×				
MER89-int		×							
MIR								×	×
MIRb								×	×
MLT1A0						×			×
MLT1J									×
MLT1L									×
MLT2D							×		
MSTA-int				×	×				
REP522		×	×						
